# Efficacy of lutein supplements on macular pigment optical density in highly myopic individuals: A randomized controlled trial

**DOI:** 10.1097/MD.0000000000033280

**Published:** 2023-03-24

**Authors:** Takeshi Yoshida, Yasutaka Takagi, Tae Igarashi-Yokoi, Kyoko Ohno-Matsui

**Affiliations:** a Department of Ophthalmology and Visual Science, Tokyo Medical and Dental University, Tokyo, Japan; b Department of Advanced Ophthalmic Imaging, Tokyo Medical and Dental University, Tokyo, Japan; c Japan Medical Affairs, Japan business, Santen Pharmaceutical Co. Ltd., Osaka, Japan.

**Keywords:** high myopia, lutein, macular pigment, macular pigment optical density

## Abstract

Lutein supplementation is beneficial in preventing maculae from developing serious ocular diseases. This study aimed to evaluate the efficacy and safety of lutein administration in patients with high myopia (HM).

**Methods:**

In a single-center randomized double-blinded placebo-controlled trial conducted over 24 months, 22 eyes were enrolled in lutein and control groups. Among them, 15 eyes in the lutein group and 13 eyes in the control group completed the study. All patients with HM (axial length > 26.00) were administered lutein (20 mg) or placebo once daily for 6 months. The macular pigment optical density (MPOD), rate of change in MPOD, visual acuity, contrast sensitivity, and electroretinogram after administration were examined at baseline, 3 months, and 6 months.

**Results:**

The baseline MPOD in the control and lutein groups was 0.71 ± 0.21 and 0.70 ± 0.22, respectively. The MPOD in the control and lutein groups at 3 months was 0.70 ± 0.21 and 0.70 ± 0.25, respectively, and at 6 months was 0.66 ± 0.20 and 0.72 ± 0.27, respectively, which was not significantly different from those at baseline or between the groups. The MPOD significantly increased from baseline in the lutein group with less than 28.25 mm of axial length at 6 months (from 0.71 ± 0.20 to 0.78 ± 0.22, *P* = .02, *t* test). visual acuity, contrast sensitivity, and electroretinogram values were similar between the groups.

**Conclusion:**

Lutein supplementation showed significant benefits in MPOD augmentation in patients with HM.

## 1. Introduction

The macula in the retina is a yellow-pigmented area at the posterior pole of the eye that allows central vision and provides the most acute visual acuity (VA) and best color identification.^[1]^ Macular yellow pigments primarily consist of lutein and its structural isomer zeaxanthin.^[2]^ Lutein is one of the few xanthophyll carotenoids found in high concentrations in the macula. Since de novo synthesis of lutein within the human body is impossible, it can only be obtained from the diet.^[3]^ Macular pigments (MP), including lutein, are concentrated in the photoreceptor axons of the Henle nerve fiber layer and rod outer segments^[4]^ where they easily undergo oxidative attack. Lutein is a potent antioxidant. Several basic and clinical studies have reported the antioxidative and anti-inflammatory properties of lutein in the eye, suggesting that lutein plays a major role in protecting the retina and retinal pigment epithelium from light-initiated oxidative damage by scavenging reactive oxygen species and filtering blue light. Thus, lutein is involved in the putative pathogenesis of many age-related eye diseases such as age-related macular degeneration (AMD),^[5]^ retinitis pigmentosa,^[6]^ and diabetic retinopathy.^[7]^ Furthermore, lutein plays a key role in maintaining macular morphology and function.^[8,9]^

Several studies have suggested that dietary supplementation with lutein prevents eye diseases, such as AMD, and improves visual function.^[10–13]^ Feng et al^[1]^ demonstrated that supplementation with 20-mg lutein increases macular pigment optical density (MPOD). Furthermore, lutein and zeaxanthin supplementation can improve contrast sensitivity (CS),^[10]^ and low MP levels are associated with poor visual function in both healthy eyes and eyes with early AMD.^[11]^ Therefore, it is used as a key ingredient in ocular supplements.

Myopia is a serious global health concern, with an increasing prevalence among teenagers and young adults of approximately 90% in East Asia and 50% in the United States and Europe.^[12]^ High myopia (HM), characterized by progressive elongation of the eyeball (defined as an axial length [AL] > 26.0 mm), is associated with serious ocular complications such as retinal detachment, macular schisis, macular holes,^[13]^ chorioretinal atrophy, and choroidal neovascularization.^[14,15]^ Excessive elongation of the eyeball in HM results in consequential thinning of the retina, including the macula, which induces HM-related complications, for which there are no effective treatment options. In the AL > 26 mm group, a significant inverse correlation was reported between MPOD and AL.^[16]^ in vivo measurements of retinal thickness using optical coherence tomography revealed an inverse relationship between AL and macular thickness.^[17–20]^ This is the reason for the low MPOD in HM because MP, including lutein, is concentrated in the photoreceptor axons of the Henle nerve fiber layer and rod outer segments.^[4]^ However, the advantages of high MP and the efficacy of lutein supplementation in patients with HM remain uncertain, although MP decreases in eyes with HM.

Most ocular complications of HM are irreversible and it is difficult to recover the VA and visual field; hence, therapeutic options are required to prevent ocular complications. Lutein supplementation improves visual function in patients with AMD,^[10]^ and studies have explored whether dietary supplementation with lutein might prevent AMD or improve the condition of patients with AMD.^[14,21,22]^ The efficacy of lutein supplementation in patients with HM remains unclear, and to our knowledge, no studies have been conducted in these patients with HM. Therefore, we aimed to evaluate the efficacy and safety of lutein supplementation in HM patients.

## 2. Methods

### 2.1. Design overview

This single-center, randomized, double-blind, placebo-controlled study was conducted between September 2017 and March 2021. We used placebo as a control to evaluate the efficacy and safety of lutein in patients with HM. The main components of the placebo were safflower oil and a stirring agent that had no drug effect. In total, 44 patients were enrolled in the trial in either the intervention or control group (ratio 1:1). All patients were administered the investigational drug or placebo orally, once daily for 6 months, and were interviewed during follow-up visits 3 and 6 months after the initiation of drug administration to complete the efficacy and safety assessments.

The study design was approved by the ethics committee of Tokyo Medical and Dental University. All patients were informed of the possible benefits and risks of the study, and provided signed informed consent. This study was performed in accordance with the ethical standards of the 1964 Declaration of Helsinki and its later amendments, and the Clinical Trials Act in Japan. This study was registered as a clinical trial in the Japan Registry of Clinical Trials (jRCT; ID: jRCTs031180168).

### 2.2. Setting and participants

Patients were recruited from the Tokyo Medical and Dental University Hospital. The inclusion criteria were as follows: Japanese origin, AL of eyeball 26.5 mm or more, and less than 30.0 mm; age 20 to 50 years, and patient consent. The exclusion criteria were as follows: comorbid severe primary diseases, such as cardiovascular disease, cerebrovascular disease, and diseases of the liver, kidneys, or hematopoietic system; pupil diameter less than 6.5 mm in mydriasis; vision-affected ocular diseases, such as glaucoma, AMD, and diseases of the retina; administration of lutein within the last 6 months; allergy to multiple drugs or known allergy to lutein capsule components; and other conditions considered unsuitable for participation after discussion.

### 2.3. Randomization and masking

Stratified random numbers were generated by statisticians using the SAS version 9.3 software (SAS Institute Inc., Cary, NC), which denoted the drug number as a running serial number for the sample size. The investigator administered the corresponding drugs to participants according to the sequence in which they were enrolled.

Unified manufacturing of the study drugs (lutein and placebo) was conducted by Santen Pharmaceutical Co. Ltd. Patients in the lutein group were orally administered lutein, 1 capsule, once daily (20 mg/capsule), whereas those in the placebo group were orally administered placebo identical to that in the lutein group daily. The treatment duration was 6 months. There were 2 levels of blinding: first, blinding of each drug to its corresponding group; and second, blinding of the drug was conducted by the blinding staff after the blinding status examination and completion of data locking. The second round of unblinding was performed after the completion of the statistical analysis and unmasking of the placebo and experimental groups. All participants, investigators, and staff members who performed the analyses were blinded to the study protocols.

### 2.4. Outcomes and measurements

The primary efficacy marker was the change in MPOD after 6 months of administration relative to that at baseline. To improve data accuracy, the MPOD was evaluated at least 3 times. The secondary efficacy markers were change in VA, change in CS, and change in electroretinogram (ERG) measurements (all relative to baseline values). The safety marker was the presence or absence of adverse events.

### 2.5. Measurement of macular pigment optical density

The MPOD measurements were performed using a standardized protocol based on the psychophysical method of heterochromatic flicker photometry. This protocol had high test–retest reliability (*R* = 0.9), and the participant responses at the 2 wavelengths were consistent with the absorption spectrum of lutein.^[15,17,18,23]^ Briefly, participants were fitted with trial frames and appropriate lenses for testing after refraction. The optimal flicker frequency for heterochromatic flicker photometry was determined for each participant. Measurements were made with the eye at the foveal center, and the participant made 4 separate determinations. The participants viewed a small test field superimposed on a blue background with their right eye. The test field alternated between a wavelength (blue or blue-green) that was absorbed by the MP and a reference wavelength (green to yellow-green) outside the absorption band of the MP. The test field appeared to be flicker when the frequency of alternation was chosen correctly. While performing the measurements, the participants were instructed to adjust the energy of the bluish test light to stop flickering. The amount of bluish light required to nullify the flicker provides a measure of the absorption of MP (i.e., MPOD) at the retinal location of the test light. The participants were instructed to fixate on the center of the target.

### 2.6. Visual acuity

The VA of the patients was measured with the best refractive correction using the Nidek system Chart SC-1600 (Nidek Co., Aichi, Japan). The best-corrected visual acuity (BCVA) was compared before and after therapy. VA was converted to the logarithm of the minimal angle resolution and analyzed.

### 2.7. Contrast sensitivity

The CS was measured using a CSV-1000E contrast testing instrument (Vector Vision, Greenville, OH) at a distance of 2.4 m under standard brightness (85 cd/m^2^). This test consisted of the following 4 spatial frequencies: 3, 6, 12, and 18 cycles per degree (cpd). If the eye showed a refractive error, refractive correction was performed during the CS test.

### 2.8. Electroretinogram

Full-field ERGs were obtained. The ERG was performed according to the standards of the International Society for Clinical Electrophysiology of Vision.^[19]^ The ERG was elicited using a light stimulator and recorded using a contact lens electrode (LE4000; Tomey, Japan). Possible correlations between the amplitudes of the a- and b-waves of the dark-adapted ERGs elicited by 200 cds/m^2^ and the MPOD were analyzed.

### 2.9. Datasets

The per-protocol set (PPS) was defined a priori in the protocol; therefore, analyses were performed on the PPS for lutein efficacy. For PPS, we excluded patients with poor compliance to the study protocol and those who refused to administer lutein tablets after baseline. In addition, the MPOD value was investigated using axial length-stratified analysis.

### 2.10. General analytical principles

Statistical analyses were performed using the SAS 9.3 software. The statistical description of quantitative data included the number of patients, mean, standard deviation, median, maximum, minimum, test statistic, and *P* value. The statistical description of the qualitative data included the frequency distribution, composition ratio, test statistics, and *P* value. Suitable statistical analysis methods were used for the intergroup and intragroup comparisons. All statistical tests were performed to obtain the test statistics and corresponding *P* values. A paired *t* test was used to directly obtain *P* values. All statistical tests were 2 tailed. Statistical significance was set at *P* < .05.

## 3. Results

### 3.1. Patient characteristics

**Figure 1.**
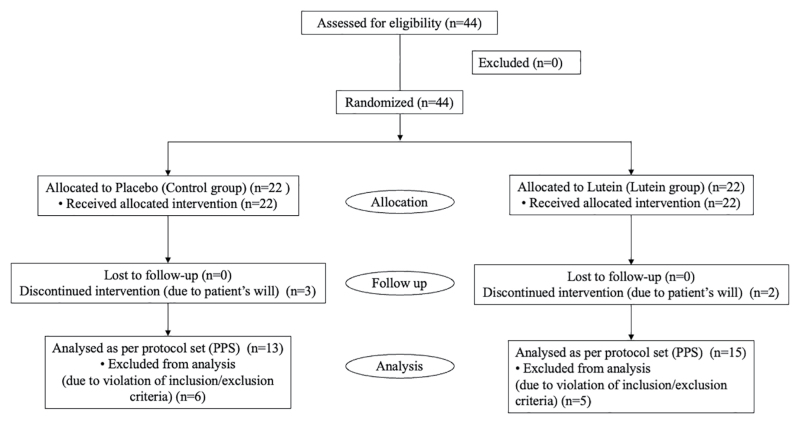
Diagram of study design.

Of the initially enrolled 44 patients with HM (n = 44 eyes), with 22 patients in the lutein and control groups, 5 refused to continue the study, and 11 were excluded. Thus, 28 patients (n = 28 eyes) completed the study as PPS, with 15 (n = 15 eyes) in the lutein group and 13 (n = 13 eyes) in the control group. The participant distribution flowchart is shown in Figure 1. The mean ages in the lutein and control groups were 46.54 ± 3.48 years and 42.80 ± 6.55 years, respectively. There were 3 men and 12 women in the lutein group and 1 man and 12 women in the control group. The average of AL, BCVA, and MPOD in the lutein and control groups were 28.21 ± 1.01 mm and 28.83 ± 0.98 mm, −0.01 ± 0.13 and −0.09 ± 0.09, and 0.70 ± 0.22 and 0.71 ± 0.21, respectively. There were no significant differences in age, sex, AXL, BCVA, or MPOD between the lutein and control groups at the baseline. Furthermore, there were no significant differences between the lutein and control groups at baseline in the CS and ERG (a-wave, b-wave, and b/a ratio). The results are presented in Table 1 presents the results.

**Table 1:** Baseline demographic and clinical characteristics using the PPS.

Maker		Control group (N = 13)	Lutein group (N = 15)	*P* value
Age (yr)		46.54 (3.48)	42.80 (6.55)	.08
Sex	Male	1	3	.60
	Female	12	12	
Axial length		28.83 (0.98)	28.21 (1.01)	.11
Visual acuity (LogMAR)		−0.09 (0.09)	−0.01 (0.13)	.08
MPOD		0.71 (0.21)	0.70 (0.22)	.92
Contrast sensitivity	3	4.31 (2.21)	3.87 (1.96)	.58
	6	3.46 (2.03)	2.80 (1.61)	.35
	12	2.77 (2.65)	2.33 (1.76)	.61
	18	2.15 (1.77)	2.80 (1.57)	.32
ERG	a wave	290.17 (39.04)	272.05 (69.85)	.42
	b wave	358.30 (67.56)	362.10 (78.69)	.89
	b/a ratio	1.27 (0.15)	1.36 (0.21)	.18

Data represent as mean (standard deviation).

ERG = electroretnogram, logMAR = logarithm of minimal angle resolution, MAR = minimum angle of resolution, MPOD = macular pigment optical density, PPS = per protocol set.

### 3.2. Changes after treatment

The MPOD values are listed in Table 2. After 3 and 6 months of treatment from baseline, the changes in MPOD in the lutein and control groups were 0.00 ± 0.20 and 0.00 ± 0.12 at 3 months and 0.03 ± 0.12 and −0.07 ± 0.13 at 6 months, respectively, with no significant differences between the groups. There were no significant differences in the baseline values. The rate of changes in the lutein and control groups were 0.00 ± 0.20% and 0.02 ± 0.20%, respectively with no significant differences between the groups.

**Table 2:** MPOD scores in the 2 groups before and after drug administration using the PPS.

	Control group (N = 13)	Lutein group (N = 15)	*P* value
MPOD scores comparison of the change			
Months 3 after treatment minus baseline	0.00 (0.12)	0.00 (0.20)	.95
Months 6 after treatment minus baseline	−0.04 (0.09)	0.03 (0.12)	.10
MPOD scores comparison of the rate of change			
Months 3 after treatment minus baseline, %	0.02 (0.20)	0.00 (0.28)	.87
Months 6 after treatment minus baseline, %	−0.07 (0.13)	0.02 (0.25)	.26

Data are expressed as mean (standard deviation).

MPOD = macular pigment optical density, PPS = per protocol set.

The BCVA (logarithm of minimal angle resolution) values are presented in Table 3. After 3 and 6 months of treatment from baseline, the changes in BCVA in the lutein and control groups were 0.01 ± 0.10 and 0.04 ± 0.07 at 3 months and 0.01 ± 0.10 and 0.04 ± 0.08 at 6 months, respectively with no significant differences between the groups.

**Table 3:** LogMAR visual acuity in the 2 groups before and after drug administration using the PPS.

LogMAR comparison of the change	Control group (N = 13)	Lutein group (N = 15)	*P* value
Months 3 after treatment minus baseline	0.04 (0.07)	0.01 (0.10)	.35
Months 6 after treatment minus baseline	0.04 (0.08)	0.01 (0.10)	.35

Data are expressed as mean (standard deviation).

logMAR = logarithm of minimal angle resolution, MAR = minimum angle of resolution, PPS = per protocol set.

The CS values are listed in Table 4. After 3 months of treatment, 3, 6, 12, and 18 cpd of the CS change from baseline in the lutein and control groups were 0.80 ± 1.82 and 0.23 ± 2.95, −0.27 ± 1.71 and −0.38 ± 2.81, −0.27 ± 2.66 and −0.15 ± 2.76, and −0.73 ± 1.67 and 0.62 ± 1.71, respectively. The differences between the 2 groups were not significant at 3, 6, and 12 cpd, but at 18 cpd (*P* = .05). After 6 months of treatment, 3, 6, 12, and 18 cpd of the CS change from baseline in the lutein and control groups were 0.47 ± 2.72 and −0.08 ± 3.23, −0.40 ± 2.85 and −0.69 ± 3.54, 0.00 ± 3.23 and −0.15 ± 2.64, and −0.53 ± 2.26 and 0.23 ± 2.01, respectively. The differences between the 2 groups were not significant at any point.

**Table 4:** Contrast sensitivity in the 2 groups before and after drug administration using the PPS.

Contrast sensitivity comparison of the change		Control group (N = 13)	Lutein group (N = 15)	*P* value
Months 3 after treatment minus baseline	3	0.23 (2.95)	0.80 (1.82)	.54
	6	−0.38 (2.81)	−0.27 (1.71)	.89
	12	−0.15 (2.76)	−0.27 (2.66)	.91
	18	0.62 (1.71)	−0.73 (1.67)	.05
Months 6 after treatment minus baseline	3	−0.08 (3.23)	0.47 (2.72)	.63
	6	−0.69 (3.54)	0.40 (2.85)	.37
	12	−0.15 (2.64)	0.00 (3.23)	.89
	18	0.23 (2.01)	−0.53 (2.26)	.36

Data are expressed as mean (standard deviation).

PPS = per protocol set.

The rate of change in the amplitude of the a-wave (μV), b-wave (μV), and ratio of a/b-wave in the ERG are listed in Table 5. After 3 months of treatment, the rate of change in the a-wave, b-wave, and ratio of a/b-wave from baseline in the lutein and control groups was −0.03 ± 0.25 and 0.06 ± 0.22, −0.02 ± 0.19 and 0.08 ± 0.17, and 0.03 ± 0.15 and 0.04 ± 0.26, respectively, and the differences between the 2 groups were not significant for each factor. After 6 months of treatment, the rate of changes in the a-wave, b-wave, and ratio of a/b-wave from baseline in the lutein and control groups was 0.00 ± 0.26 and 0.04 ± 0.20, −0.02 ± 0.23 and 0.04 ± 0.23, and −0.02 ± 0.14 and −0.01 ± 0.10, respectively, and the differences between the 2 groups were not significant for each factor.

**Table 5:** ERG scores in the 2 groups before and after drug administration using the PPS.

ERG scores comparison of the rate of change		Control group (N = 13)	Lutein group (N = 15)	*P* value
Months 3 after treatment minus baseline, %	a wave	0.06 (0.22)	−0.03 (0.25)	.32
	b wave	0.08 (0.17)	−0.02 (0.19)	.15
	b/a ratio	0.04 (0.26)	0.03 (0.15)	.93
Months 6 after treatment minus baseline, %	a wave	0.04 (0.20)	0.00 (0.26)	.69
	b wave	0.04 (0.23)	−0.02 (0.23)	.52
	b/a ratio	−0.01 (0.10)	−0.02 (0.14)	.81

Data are expressed as mean (standard deviation).

ERG = electroretnogram, PPS = per protocol set.

### 3.3. Stratified macular pigment optical density analysis based on axial length

MPOD outcomes were further stratified using different AL strategies (Table 6). The participants were classified into 2 groups according to AL ≤ 28.25 and >28.25 mm. In the lutein group, 9 eyes had AL ≤ 28.25 mm and 6 had AL > 28.25 mm AL. In the control group, 4 eyes had an AL ≤ 28.25 mm and 9 had an AL > 28.25 mm.

**Table 6:** MPOD scores in the Lutein groups before and after Lutein administration using the PPS.

MPOD scores	AL < 28.25 mm			AL ≥ 28.25 mm		
	Control group (N = 4)	Lutein group (N = 9)	*P* value	Control group (N = 9)	Lutein group (N = 6)	*P* value
Baseline	0.67 (0.25)	0.71 (0.17)	.69	0.72 (0.21)	0.67 (0.29)	.70
Comparison of the change						
Months 3 after treatment minus baseline	−0.04 (0.20)	0.06 (0.17)	.35	0.02 (0.08)	−0.09 (0.22)	.22
Months 6 after treatment minus baseline	−0.09 (0.05)	0.09 (0.10)	.01	−0.02 (0.10)	−0.07 (0.07)	.35
Comparison of the rate of change						
Months 3 after treatment minus baseline, %	−0.02 (0.30)	0.09 (0.23)	.46	0.03 (0.14)	−0.14 (0.27)	.14
Months 6 after treatment minus baseline, %	−0.12 (0.04)	0.13 (0.15)	.01	−0.03 (0.13)	−0.17 (0.23)	.16

Data are expressed as mean (standard deviation).

AL = axial length, PPS = protocol per set.

In the AL ≤ 28.25 mm subgroups, the average of AL at baseline was 27.50 ± 0.46 mm in the lutein group and 27.66 ± 0.59 mm in the control with no significant difference between the groups. After 3 months from baseline, the change in MPOD in the lutein and the control group was 0.06 ± 0.17 and −0.04 ± 0.20, respectively, with no significant difference between the groups. The rate of MPOD changes in the lutein and the control group was 0.09 ± 0.23% and −0.02 ± 0.30%, respectively with no significant difference between the groups. In contrast, after 6 months, the changes in MPOD from baseline in the lutein and control groups were 0.09 ± 0.10 and −0.09 ± 0.05, respectively, and the rate of MPOD changes from baseline in the lutein and control groups was 0.13 ± 0.15% and −0.12 ± 0.04%, respectively, with a significant difference in both the values and rate (*P* = .01, *P* = .01, respectively). In the AL > 28.25 mm subgroups, the average of AL at baseline was 29.27 ± 0.53 mm in the lutein group and 29.35 ± 0.54 mm in the control with no significant difference between the groups. There were no significant differences between the groups either in the change in MPOD or in the change in rates after 3 and 6 months from baseline. In the stratified analysis, we also evaluated BCVA, CS, and ERG; however, we found no significant changes in the baseline values for each parameter.

### 3.4. Adverse events

None of the patients reported any adverse events or complications.

## 4. Discussion

In this RCT, we evaluated the effects of lutein supplementation on MPOD. We observed that lutein supplementation significantly increased the MPOD level over 6 months and might be beneficial in preventing the loss of macular pigments in patients with HM having <28.25 mm of AL. To our knowledge, this is the first prospective, randomized, double-blind, placebo-controlled clinical trial to confirm the clinical efficacy and safety of lutein administration in HM patients.

HM is a clinical risk factor of serious ocular complications. The complications increase proportionally with an increase in AL.^[14,15,22]^ HM-related complications are often observed in the macular areas. Clinically, there appears to be a myopic maculopathy pattern. It ranges from the early appearance of a tesselated fundus to progressive development of diffuse atrophy and lacquer cracks, followed by progression to patchy atrophy. Choroidal neovascularization (CNV) generally develops adjacent to areas of patchy atrophy or lacquer cracks, resulting in irreversible and severe VA loss. VA in HM may be subnormal, even before advanced myopic maculopathy sets in. One of the reasons for this may be the alteration in the arrangement of photoreceptors, which is affected by excessive stretching of the posterior pole. This may lead to a subnormal visual function in the macula. In eyes with HM, the photoreceptors in the nasal hemiretina are aligned toward the optic nerve by eyeball stretching and the retina becomes thin. MPs are mainly distributed in the Henle fiber layer of the macular fovea and the outer ganglion layer of photoreceptors in the rod cells around the fovea.^[20]^ Therefore, the concentration of MP in the fovea of HM is thought to be low due to retinal thinning. A previous study showed that MPOD was negatively correlated with the degree of myopia.^[24]^ In this study, we showed that the average MPOD in patients was approximately 0.70 at baseline. Zhang et al^[24]^ showed that the MPOD in HM with an of 27.87 mm of AL was 0.55which is similar to our results. MPOD positively correlated with central foveal thickness in patients with low-to-moderate myopia.^[25]^ In the stratified analysis by AL of HM eyes in our study, we showed that MPOD values after supplementation significantly increased in individuals with HM with AL ≦ 28.25 mm, which is in line with previous reports because the retinal thickness in HM becomes thinner with AL elongation and the degree of myopia. Retinal thickness may also be an important factor when considering the efficacy of lutein supplementation in HM. However, the efficacy of lutein supplementation in patients with HM remains controversial, although the benefits may be dose dependent. Tanito et al^[26]^ found that MPOD levels after 3 months of lutein supplementation did not increase in individuals with myopia exceeding −4 diopters. They were orally administered 10 mg lutein for 3 months. In this study, we orally administered 20 mg lutein for 6 months, and the MPOD level did not increase at 3 months; however, it significantly increased at 6 months in individuals with HM and AL ≤ 28.25 mm. Another study demonstrated that low-dose lutein supplementation (6 mg) did not significantly improve the MPOD in patients with early AMD.^[27]^ Furthermore, Sasamoto et al^[27]^ observed that daily supplementation with 6 mg lutein did not affect the MPOD level in healthy individuals over 1 year, and speculated that 6 mg lutein may be insufficient to increase the MPOD level. Thus, high-dose and long-term administration may be required to increase the MPOD in eyes with or without HM.

A decrease in MP is related to functional abnormalities of the macula, which eventually lead to age-related degenerative eye diseases.^[28,29]^ It is hypothesized that carotenoids, including lutein, could protect the photoreceptors and the retinal pigment epithelium by screening these susceptible retinal structures for actinic blue light and quenching reactive oxygen species.^[30]^ Barker et al^[31]^ demonstrated that carotenoid supplementation resulted in the accumulation of MP and significant foveal protection against short-wavelength photochemical damage. In humans, dietary lutein and zeaxanthin intake is inversely associated with AMD risk.^[32–34]^ In addition, Ma et al^[35]^ found that supplementation with these macular carotenoids partially reversed the loss of visual function in patients with early AMD by elevating MPOD, suggesting a causative role of MPOD in the maintenance of normal visual function. Thus, some studies have suggested the importance of MP in protecting visual function from age-related damage.^[36–38]^ However, whether lutein supplementation improves visual function remains controversial. We showed that the MPOD increased in HM individuals with AL less than 28.25 mm, and their visual functions, including VA, CS, and ERG, were maintained, but did not improve compared with that at baseline. Moreover, MPOD was not increased in the HM eyes with AL > 28.25 mm by lutein supplementation. One possibility is retinal thinning in the eyes with HM. Lutein is concentrated in the photoreceptor axons of the Henle nerve fiber layer and rod outer segments of the retina.[4](34)[4](34)^[4,39]^ Furthermore, we observed that the MPOD of the HM eye is low because the retinal thickness of HM eyes is extremely thin owing to extreme eyeball elongation, indicative a lack of space for MP accumulation in retina. Another possible explanation could be the duration of lutein administration. Greater MP accumulation may be required to improve the visual function in eyes with HM. In the present study, patients with HM were administered lutein supplements for 6 months, and the MPOD was increased in the eyes with less than AL < 28.25 mm with the supplementation; however, visual functions were not improved. Longer administration of lutein may induce further MP accumulation, and it may be possible to be improved visual function in HM eyes. Further investigation with longer observation is necessary for a complete understanding of the association between the MPOD increase by lutein and the visual function in HM eyes.

Although myopia is not often considered a serious eye disorder because vision can be easily corrected with glasses or contact lenses, it increases the risk of other ocular pathologies, such as glaucoma, retinal detachment, and lacquer cracks.^[40]^ A low level of MPOD may predispose myopic patients to lacquer cracks,^[16]^ which is one of the complications of high myopia characterized by rupture of Bruch’s membrane and retinal pigment epithelium.^[16,41]^ A previous report noted that eyes with lacquer cracks around the macula have a higher risk of developing myopic CNV, which is one of the most serious complications in HM eyes.^[41,42]^ Furthermore, a cross-sectional demonstrated approximately 40% reduced odds of myopia (OR = 0.57) among individuals with the highest lutein concentration of 20% in plasma.^[43]^ Thus, lutein supplementation may have the potential to prevent ocular complications, such as CNV development, and restore visual function in HM eyes. Lutein is generally regarded as safe, with minimal side effects associated with its long-term consumption. Currently, lutein is commercially available and consumed by many people worldwide. No side effects of lutein supplementation were observed in our study. Lutein supplementation has been extensively researched and is the current clinical standard for treating individuals at a risk of AMD. In addition, when lutein supplementation was coupled with omega-3 supplementation, carotenoid bioavailability was enhanced.^[44,45]^ In the present study, we observed no development of myopic CNV in the both group during the administration; however, it still remains unclear about the protective effect in the development of myopic CNV in HM eyes. Taken together with the previous studies, lutein supplementation may have a potential to reduce the risk of HM-specific ocular complications, and further investigation with longer observation is necessary for complete understanding.

This study had several limitations. First, the intervention time was relatively short (6 months) and was performed using a single dosing strategy. Long-term observation is needed to determine the functional effect of lutein because HM usually occurs in childhood and gradually develops over the long term, sometimes even >50 years. However, it is uncertain whether a higher dosing strategy would provide greater benefits. A previous trial illustrated the role of nutritional supplementation in maintaining lutein levels in the blood and MPOD, and clarified its safety in normal subjects.^[46]^ Second, our cohort had a relatively small sample size, which reduced the statistical power to assess the association with MPOD supplementation. Third, other variables such as dietary supplementation with carotenoid-rich foods were not regulated in this study. Further research is needed to study the association between different responses to dietary supplementation with carotenoid-rich foods.

## 5. Conclusions

To the best of our knowledge, this is the first randomized clinical study to assess the benefits of lutein supplementation in highly myopic individuals. Our findings showed significant benefits of lutein supplementation in MPOD augmentation in patients with HM. As this study included patients who underwent 6 months of follow-up, this might limit the evaluation of the extended effects. Further larger-scale and longer-term studies are required to strengthen these associations and evaluate the effects of lutein on visual function in patients with HM.

## Acknowledgments

The authors would like to thank Aiko Ibuki, Natsumi Miyagawa, and Chihiro Ono for MPOD examination and data collection.

## Author contributions

**Conceptualization:** Takeshi Yoshida, Yasutaka Takagi, Kyoko Ohno-Matsui.

**Data curation:** Takeshi Yoshida, Tae Igarashi.

**Formal analysis:** Takeshi Yoshida.

**Funding acquisition:** Takeshi Yoshida.

**Investigation:** Takeshi Yoshida.

**Methodology:** Takeshi Yoshida, Yasutaka Takagi.

**Project administration:** Takeshi Yoshida.

**Resources:** Takeshi Yoshida.

**Software:** Takeshi Yoshida, Tae Igarashi.

**Supervision:** Takeshi Yoshida.

**Validation:** Takeshi Yoshida, Tae Igarashi.

**Visualization:** Takeshi Yoshida.

**Writing – original draft:** Takeshi Yoshida.

**Writing – review & editing:** Takeshi Yoshida, Yasutaka Takagi, Kyoko Ohno-Matsui.
